# The Detection and Analysis of Microplastics in a Typical Mountainous Drinking Water System in China

**DOI:** 10.3390/toxics12110807

**Published:** 2024-11-08

**Authors:** Chaoxing Xu, Lingzhen Pan, Linfu Zhai, Wenlong Wang, Kejia Lu, Jianqiang Zhu, Guanghua Xia

**Affiliations:** 1 Institute of Environmental Engineering Technology, School of Life Sciences, Taizhou University, Taizhou 318000, China; xuchaoxing0903@163.com (C.X.); lukejiawork@163.com (K.L.); zhujq@tzc.edu.cn (J.Z.); 2 Taizhou Environmental Science Design and Research Institute Co., Ltd., Taizhou University, Taizhou 318000, China; 3 Taizhou Jinghe Testing Technology Co., Ltd., Taizhou 318000, China; woshigunge@163.com (L.P.); 15824098150@163.com (L.Z.); 4 Zhejiang Taicheng Environmental Technology Co., Ltd., Taizhou 318000, China; 15258595998@163.com

**Keywords:** microplastic, mountainous area, drinking water, daily intake, human exposure

## Abstract

Microplastics (MPs) are widely detected in urban drinking water systems. However, the presence and characteristics of MPs in mountainous drinking water systems with independent and simple filtration facilities have been overlooked. In this study, we revealed the ubiquity of MPs and demonstrated that their concentrations increased along with the pipeline length in Bainitan Village, Tiantai County, China. The simple filtration facility in this village did not effectively remove most MPs. Polyethylene, polyurethane, and polyethylene terephthalate were the dominant polymers in water samples (72.32% in total), while polyvinylchloride, polyurethane, and polyethylene were the most prevalent in the sediment (74.00% in total) of the reservoir. Long fragments were the predominant shape of MPs in all samples, with the majority being smaller than 100 μm. The estimated daily intake of MPs through drinking water ingestion was highest in infants (2.14–31.26 MPs/kg bw/day), compared to children (1.41–20.67 MPs/kg bw/day) and adults (1.05–15.35 MPs/kg bw/day), highlighting their increased vulnerability. This emphasizes the need for advanced water treatment systems in mountainous regions. It also underscores the necessity for government attention to improve water safety in remote areas. Our research will contribute valuable baseline data for further research on MP exposure, particularly in mountainous communities.

## 1. Introduction

Plastic items are durable and cost-effective, leading to a global increase in their production annually, reaching 390 million tons in 2021 [1,2,3]. Due to these advantages, plastics are now widely used in daily life, enhancing convenience in many fields [4]. The sustainable growth of plastic production continues, despite significant concerns raised by governments and social organizations [5]. These plastic products release numerous smaller waste items into the environment through fragmentation and degradation [5]. Plastic debris smaller than 5 mm, known as microplastics (MPs), was first defined by [6]. Because MPs are difficult to degrade and easy to transfer in the environment, they are now ubiquitous in various environmental matrices, including water, soil, sediments, and dust [7,8,9]. They have been detected in organisms such as insects, fish, birds, and even human tissue [10,11,12,13]. MPs can transfer along the food chain, due to their hydrophobicity [14]. Studies have shown that MPs cause immunoreactions, induce lung injury, affect reproductive function, and damage the digestive system in humans and other organisms [15,16,17,18]. MPs can also release pollutants (e.g., heavy metals, microorganisms, and persistent organic pollutants) adsorbed on their surface, exerting adverse health effects [19,20,21,22].

It is clear that people worldwide are generally exposed to MPs in various ways [23,24,25]. Drinking water is a significant source of microplastic intake for humans [26]. Tong et al. [27] reported that an average of 440 MPs would be ingested if a person consumed 1 L of tap water from China per day. In China, most drinking water treatment plants (DWTPs) are set up for urban and rural area residents, with water sources usually being large reservoirs near cities [28]. Rivers and lakes that serve as sources of drinking water may be several possible sources of human exposure to MPs [11,29,30].

Seven types of MPs, mainly polyethylene (PE, 26.8%), polypropylene (PP, 13.2%), polystyrene (PS, 16.5%), and polyethylene terephthalate (PET, 16.1%), were detected in raw water samples in Changsha, China, with a mean concentration of approximately 2753.00 MPs/L. Mean concentrations in treated water and tap water were 351.90 and 343.50 MPs/L, respectively [7]. Li et al. [31] revealed that the mean abundance of MPs in raw water ranged from 12.80 to 25.07 MPs/L in Zhengzhou, China. Polyvinyl chloride (PVC), polyphenylene oxide (PPO), and PET were detected as prevalent polymer types.

Several studies demonstrated that treatment stages (e.g., coagulation-flocculation-sedimentation, sand filtration, and membrane filtration) of DWTPs can remove MPs. Ma et al. [32] found that using a coagulant at a 13.5 mg/L aluminum salt dosage could remove 8.28% ± 1.06% of polyethylene MPs smaller than 0.5 mm. However, small-size MPs do not settle well because they do not bind well to floccules, exhibiting a low removal efficiency by the coagulation–flocculation–sedimentation process [33]. It was reported that most (97%) MPs in wastewater are removed by adhering to the surface of the sand grains in the rapid sand filtration process. The sieving, trapping, and adsorption effects play key roles in this MP removal process [34,35,36]. Della Rocca et al. [37] demonstrated that MPs are stopped by pores of the membranes with smaller sizes and retained on the membrane surface. However, tap water is conveyed to users through drinking water distribution systems (DWDSs), including pipelines, pumps, valves, etc. Steel, ductile iron, and plastic are common materials in DWDSs [38,39]. Researchers suggested that MPs in the distribution systems are increasing due to mechanical wear and aging of the pipes [40]. Additionally, the MPs in pipe scales may be released into the tap water, raising the MP content at the terminal of DWDSs [41]. A study showed that average microplastic (MP) abundances were 47.00 and 0.90 ± 1.30 MPs/L in tap water in Finland and Saudi Arabia, respectively [42,43]. Another study demonstrated that 9.18 MPs/L of MP were detected in tap water in the USA, which is higher than in less developed countries (e.g., Cuba, Ecuador, Uganda) [44]. Many people live in mountainous areas of China, especially in the Zhejiang Province. However, most relatively large-scale DWTPs were established for urban residents. Many studies have investigated the fate of MPs in DWTPs in towns and cities [42,43,44], while rural areas have attracted less notice, especially mountainous regions (without access to the urban tap water system) with simple drinking water treatment devices and simple DWDSs.

In this study, water samples (n = 9) from the local reservoir to tap and sediment samples from the same reservoir (n = 3) were collected in the mountainous areas of Taizhou, China and analyzed for MPs (Figure S1). We examined and compared the abundances, sizes, and chemical compositions of MPs in these samples from different sampling sites. Finally, the daily intakes of MPs via drinking water ingestion for infants, toddlers, children, and adults were estimated. As far as we know, we are the first to report the differences in MP features in drinking water in isolated villages with simple DWTPs. The results of this study are important for understanding the current state of MP contamination in drinking water supply systems in mountainous areas and evaluating the ingestion risk of MPs to humans.

## 2. Materials and Methods

### 2.1. Study Area and Sample Collection

The samples in this study were collected from Bainitan Village, located in the middle of Tiantai County, Zhejiang Province, China. Bainitan Village is a small community with approximately 400 households and about 1000 residents (Figure S1). The water distribution system was constructed in 2019. There have been no documented instances of industrial waste disposal in this village.

One to three liters of water (0–30 cm in depth) were collected using a 1 L stainless-steel tank at each site and transferred to a 3 L glass bottle. The water samples were then passed through a 5 μm (47 mm) polytetrafluoroethylene (PTFE) membrane using vacuum filtration in situ. The membranes were carefully placed into a glass Petri dish and safely transported to the laboratory. Sediment samples (5–15 cm in depth) were collected using a stainless-steel shovel and transferred to a 500 mL glass bottle, with approximately 200 g of sediment per sample.

A total of 9 water samples were collected: 3 reservoir water samples, 1 water sample before the simple filtration facility (only a simple sand filter, no coagulants were added, and the flow rate was about 5 m^3^/h), 4 tap water samples, and 1 cold-boiled (5 min at 100 °C) water sample from locations W1–W3, W4, W6–W9, and W5 (same tap as W6), respectively. Three sediment samples (S1–S3) were collected from the same locations as the water sampling sites (W1–W3). All water samples (membranes with residues) and sediment samples were stored in a refrigerator at 4 °C prior to analysis. Further details regarding the samples are provided in the Supplementary Materials (Table S1).

### 2.2. Sample Treatment

We treated the MPs in water and sediment samples following the methods outlined in previous research, with certain modifications [45]. For the water samples (Figure S1), the membranes were immersed in an ethanol solution (analytically pure, Sinopharm Chemical Reagent Co., Ltd., Shanghai, China) and subjected to ultrasonic treatment, causing the particles on the membrane to disperse into the ethanol solution. The organic matter in the solution was degraded via peroxidation using 30% H_2_O_2_ (Sinopharm Chemical Reagent Co., Ltd.) for 24 h. After digestion, the solution was filtered onto a 13 μm stainless-steel membrane using vacuum filtration (JINTENG, Tianjin, China). The membrane was then submerged in a ZnCl_2_ (analytical reagent) solution and underwent ultrasonication to ensure matters on the membrane were transferred to the solution. Mud and sand were allowed to settle after standing for 27 h.

For the sediment samples, approximately 20 g of sediment was weighed into a glass beaker, mixed well with ZnCl_2_ solution for 2 min, subjected to ultrasonication for 30 min, and left undisturbed for 48 h. The supernatant was then passed through a 13 μm stainless-steel membrane using vacuum filtration. The membrane was added to a 30% H_2_O_2_ solution and left to stand for 27 h.

The suspension from the water samples and the treated solution from the sediment samples were passed through a 13 μm stainless-steel membrane using vacuum filtration. The membranes were immersed in an ethanol solution and subjected to ultrasonic treatment. Afterward, the membranes were removed from the solution and repeatedly washed with ethanol. The final ethanol solutions from both the water and sediment samples were concentrated to 150 μL using an infrared drying oven and transferred to a highly reflective glass before laser direct infrared (LDIR) spectroscopy testing. Additional treatment details are outlined in the Supplementary Materials.

### 2.3. Identification and Quantitation of Microplastics

MPs in water and sediment sample extracts were analyzed using an Agilent 8700 LDIR chemical imaging system (Waldbronn, Germany) to determine the particle material, size (20–500 μm), and abundance. Spectra for all particles within the mid-infrared range (1800–975 cm^−1^) were obtained. Referring to the study by Huang et al. [46], spectra obtained from potential MPs were compared against a standard spectra library to identify the polymer types with a high degree of match, defined as a score greater than 0.65. The abundance of identified MPs was expressed as the number of particles per liter of water or per kilogram of sediment.

### 2.4. Quality Assurance and Quality Control (QA/QC)

During the process of sample collection and laboratory treatment, all stainless-steel sieves, shovels, tin foil, and glassware (bottles, Petri dishes, and beakers) were thoroughly cleaned with pure water and ethanol before use. All instruments were cleaned with pure water and ethanol. All solvents used were filtered through a PTFE membrane (0.45 μm) before use, and glass fittings were rinsed with ethanol (Sinopharm Chemical Reagent Co., Ltd.). To reduce extraneous MP contamination, researchers wore cotton lab coats and nitrile gloves. Glassware was analyzed alongside water and sediment samples to observe any extraneous MP contamination. Samples were covered with tin foil during the entire experimental process. The results indicated that the abundance of MPs was less than 1 item per sample in the glassware.

A quality control experiment was conducted to verify the analyzing method for MPs. Approximately 30 L of pure water were vacuum-filtered through the filter membrane, and no plastic particles were found upon examination. Recovery tests were conducted, and the recovery rates of PE and PP were calculated as 93.2% and 85.0%, respectively.

### 2.5. Estimated Daily Intake Calculation

Estimated daily intake (*EDI*) of MPs for humans through drinking water ingestion was calculated by an empirical approach. Specifically, the EDI was calculated according to the following equation [25]:

$$EDI=\frac{C\times IR}{BW}$$

where C is the determined abundance of MPs in water samples (MPs/L). In accordance with the dietary guidelines for Chinese residents [47], ingestion rate (IR) means the daily ingested volume (L/day) of drinking water and was set at 1.85 (>18 years), 1.12 (3–18 years), and 0.60 (0–3 years) L/day for adults, children, and infants, respectively. Body weights (BW) were set as 60.6, 27.25, and 9.65 kg for adults, children, and infants, respectively.

## 3. Results

### 3.1. Abundance of MPs

The quality depicted in Figure S2 represents the consistency of the LDIR spectra of the identified particles and the standard sample. A quality score above 0.56 indicated a high level of confidence in the identification, and most MPs achieved this threshold.

**Figure 1:**
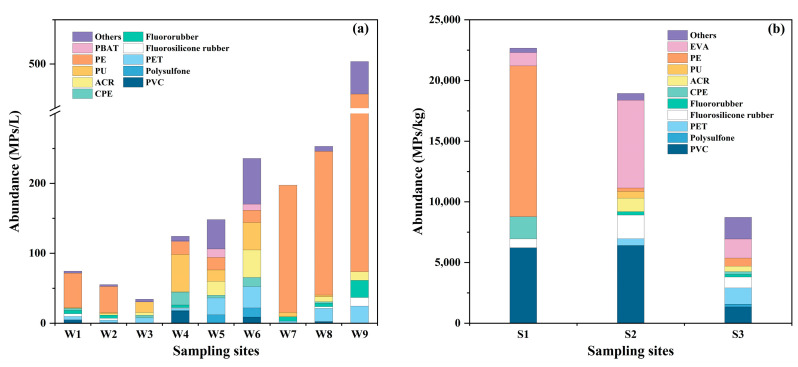
Abundances of MPs (MPs/L) in the water sample (**a**) and sediment sample (**b**) at each site.

MPs were found in all water and sediment samples in this study. The abundance of MPs in tap water (W6–W9) ranged from 197.40 to 502.82 MPs/L, with an average value of 297.23 MPs/L. The maximum MPs were found in W9 (Figure 1a). The water sampling sites W1 and W2, corresponding to S1 and S2, respectively, were located near the two main water sources for the reservoir, while W3 (S3) was situated at its outlet. The MP concentrations in the reservoir water samples were 74.51, 55.21, and 34.36 MPs/L in W1, W2, and W3, respectively (Figure 1a). The variations in MP concentrations were consistent with those found in the sediment sampling sites S1 (22,664.31 MPs/kg, dry weight), S2 (18,932.68 MPs/kg, dry weight), and S3 (8722.30 MPs/kg, dry weight), respectively (Figure 1b).

A study on the distribution of MPs in the inner lake (Tangxun Lake, Wuhan city, China) indicated that these particles tend to become embedded within the sediment layers [48]. Even in the sediments of the Amazon River, MPs have been detected, and the variation in microplastic concentration may be related to hydrological characteristics [49]. MPs, being small and lightweight, are consistently transported to reservoirs via multiple pathways, which is why they are commonly discovered in the sediment at the lakebed [50]. As an important “sink” for MPs, sediments in lakes tend to have higher concentrations of microplastics compared to the water itself [50,51,52]. Our study further corroborated this judgment. MPs in reservoirs or lakes pose a potential threat to aquatic organisms and even humans through drinking water systems [53,54]. Reservoirs serving as drinking water sources in mountainous areas deserve concern.

Many studies have shown that large-scale DWTPs for urban areas operate coagulation–flocculation–sedimentation, sand filtration, or even membrane filtration processes that can remove the majority of MPs [7,53,54]. Shen et al. [7] showed that the removal efficiency of MPs ranged from 85% to 90% in a DWTP with coagulation–sedimentation–filtration in Changsha, China. A study showed that the traditional treatment process (i.e., coagulation, flocculation-sedimentation, and filtration) could remove about 58.9–70.5% of the MPs in drinking water in an advanced DWTP in the Yangtze River Delta [55]. However, in this study, the abundance of MPs in the water sample site W4 before the simple filtration facility was 124.35 MPs/L, which was lower than that in the nearest site W6 (Figure 1a). It is evident that the simple treatment in this village failed to achieve satisfactory removal of most MPs. This implies that rural areas, especially mountainous areas, require higher-standard water treatment facilities to prevent MP pollution. Due to the significant number of people living in mountainous regions of Zhejiang or other provinces in China, the risks they face also deserve attention.

**Figure 2:**
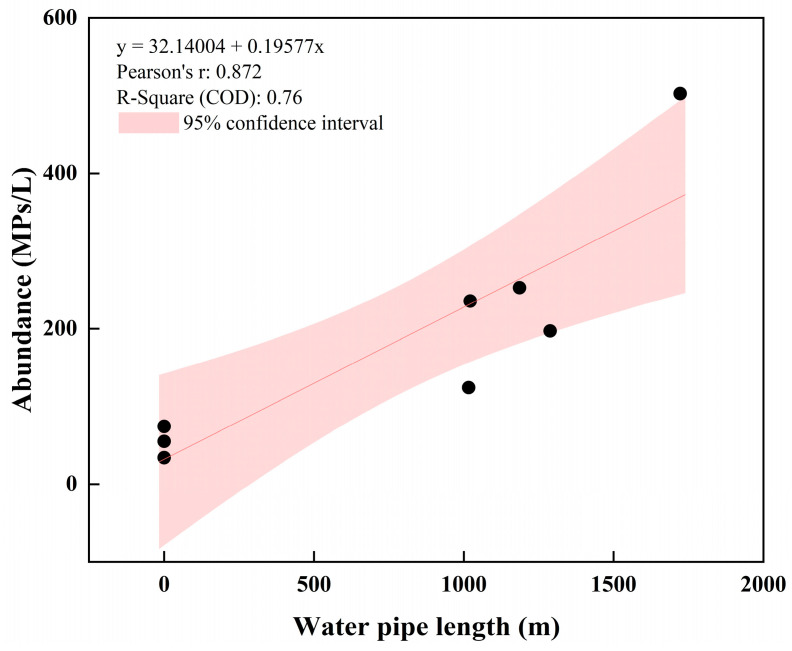
Water microplastic abundance (MPs/L) shows a significant direct correlation to the water pipe length of the simple drinking water system.

The abundance of MPs in cold-boiled water in sample site W5 (same site as W6) was 148.18 MPs/L, lower than the detected value in tap water before boiling (W6) (Figure 1a). The results were consistent with the research conducted by Yu et al. [56], who found that drinking boiled tap water reduced human ingestion of MPs. MPs in tap water were reduced by coprecipitation with calcium carbonate (CaCO_3_) incrustants in tap water upon boiling. Our research also corroborated that boiling and cooling tap water is a suggested method for rural populations to obtain safe water with fewer MPs. According to Figure 2 and Table 1, there was a clear increasing trend in water MP abundance from the whole simple water supply system as the pipe length increased. The variability in water MP abundance (MPs/L) can be well predicted (R^2^ = 0.76) using a simple linear regression (y = 32.14004 + 0.19577x). A study conducted by Yang et al. [57] pointed out that MP abundances showed a decreasing trend in DWDSs (non-plastic pipes) because MPs were broken into smaller fragments that were below the limit of detection (20 μm) or absorbed by pipe scales. However, attention should be given to the secondary release of MPs from scales in pipes [58], and it cannot be dismissed that some MPs may originate from materials utilized in the water network [59].

### 3.2. Particle Type Distribution of MPs

We identified 27 types of polymers in water samples and 17 types in sediment samples using LDIR. The abbreviations for all MPs mentioned in this study are compiled in the Supplementary Materials (Table S2). PE, polyurethane (PU), and PET were the most commonly detected polymers in water samples, whereas PVC, PU, and PE were in sediment samples (Tables S3 and S4, raw data prior to conversion into unit volumes or unit masses). These polymers collectively accounted for 72.32% and 74.00% of the total polymers in the water and sediment samples, respectively, from different sites.

**Figure 3:**
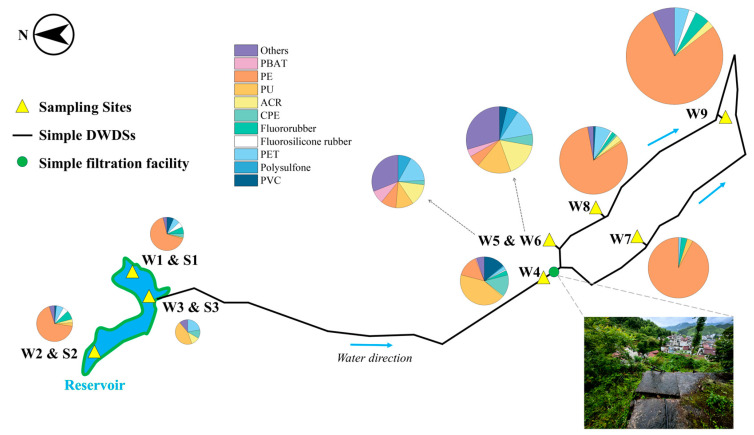
Distribution of MP types in water samples along the reservoir and DWDSs by number. (The area of the pie chart represents the quantity of MPs.).

**Figure 4:**
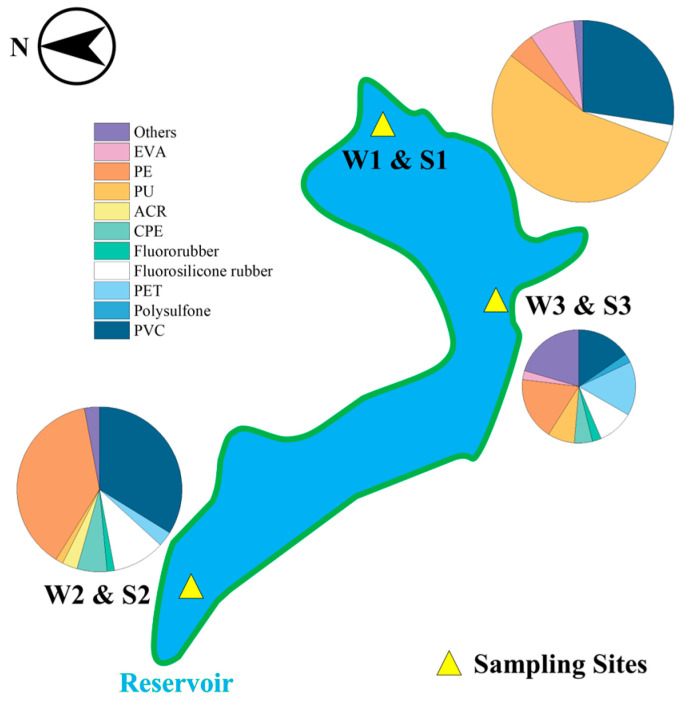
Distribution of MP types in sediment samples in the reservoir by number. (The area of a pie chart represents the quantity of MPs.).

Firstly, PVC was one of the main polymer types in sediment sample sites S1 (27.42%), S2 (33.82%), and S3 (15.38%). By contrast, the proportions of PVC in water sample sites W1, W2, and W3 were 6.49%, 1.61%, and 0.00%, respectively (Figure 1, Figure 3 and Figure 4). It suggests that PVC particles in this reservoir were prone to settling and difficult to move. Secondly, PE was the predominant polymer at sampling sites W7 (92.19%), W8 (81.31%), and W9 (78.05%), far from the reservoir, with proportions higher than those found at other sites (Figure 3). The research review indicates that MPs in water are likely due to the release of particles from the mechanical wear of plastic-coated or plastic-lined pipes or tanks [60]. A study suggested that the widespread use of plastic pipes in China’s DWSSs may contribute to MP contamination, potentially increasing the concentration of MPs (PE, PET, etc.) in tap water samples [27]. In our study area, Bainitan Village, the simple DWSSs utilized PE for their piping. Despite its durability, plastic can still undergo abrasion, which may account for the presence of specific MPs found in tap water [61]. The increase in PE particles in the water samples of this study further supports this perspective.

### 3.3. Particle Size Distribution of MPs

**Figure 5:**
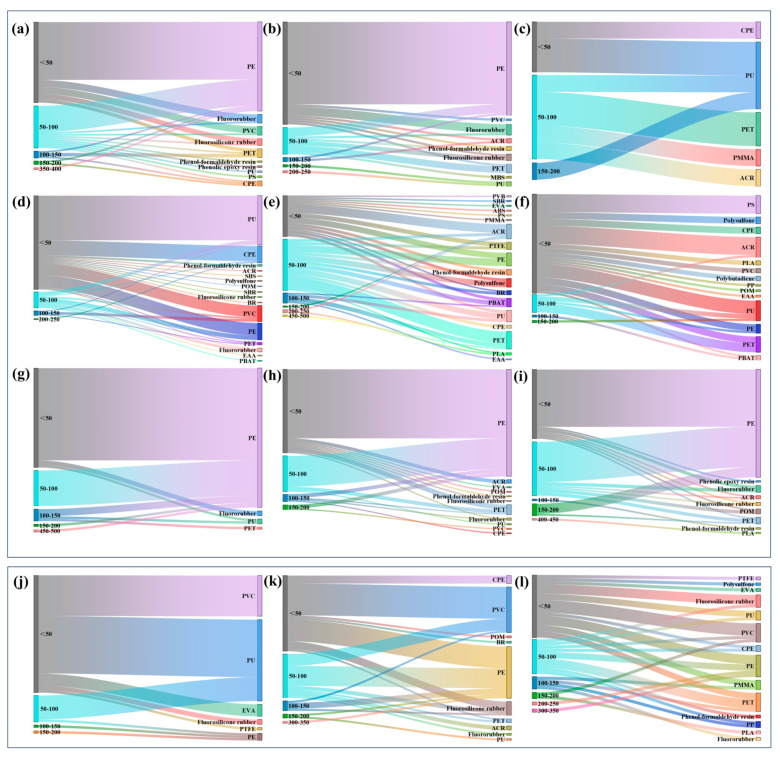
Size (μm) and MP types of MPs detected in the water samples and sediment samples. The alphabet represents the sampling site, i.e., (**a**–**l**) represent W1–W9 and S1–S3 in alphabetical order.

Figure 5 illustrates the size characteristics of MPs detected in both water and sediment samples. In our study, all detected MPs in water samples were below 500 μm in size, while those in sediment samples were below 350 μm. Among them, MPs with small sizes (100 μm) were predominant in all water samples, accounting for 90.91%, 93.55%, 88.89%, 95.35%, 85.14%, 96.30%, 89.06%, 90.65%, and 89.02% of all MPs in W1, W2, W3, W4, W5, W6, W7, W8, and W9, respectively. Extended interaction between water and polymer pipes can lead to the disintegration of the polymers into finer particles [62]. The same particle size distribution results were also observed in sediment samples S1 (96.77%), S2 (88.24%), and S3 (79.49%). Additionally, the three primary polymers in water samples, namely PE, PU, and PET, shared a common trait: the majority of them were typically less than 100 μm in size. For the three most common polymers—PVC, PU, and PE—their particles in sediment samples were also less than 100 μm in size.

**Figure 6:**
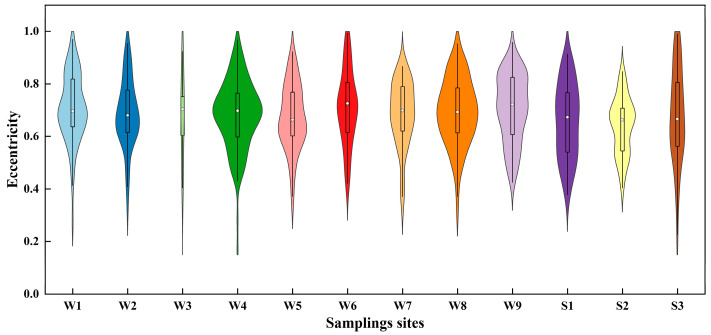
Eccentricity distributions of MPs in the water and sediment samples.

**Figure 7:**
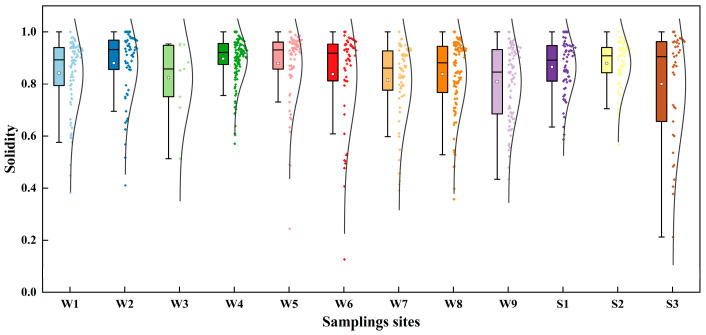
Solidity distributions of MPs in the water and sediment samples.

The eccentricity and solidity of each identified MP particle in all water and sediment samples were obtained using the Agilent Clarity software (clarity instrument 2.0). The results are shown in Figure 6 and Figure 7. Eccentricity is a standard for characterizing shape, within the scale of 0 to 1. The closer the eccentricity value is to 1, the higher the aspect ratio. This means that samples with a fibrous shape will have values close to 1, while those with a circular shape will approach 0 [46]. The solidity is the ratio of an MP particle’s area to the area of its boundary. The boundary area refers to the area of the smallest rectangle that can encompass the MP particle, with its dimensions being the height and width of the rectangle. The schematic diagram of the symbolic meaning of eccentricity and solidity is shown in Figure S3. The lower the solidity, the more closely the shape resembles that of a fiber.

Figure 6 and Figure 7 in this study reveal a scattered distribution of eccentricity and solidity among the identified MPs, reflecting the variety of shapes they exhibited. The median eccentricity of MPs in the nine water samples ranged from 0.66 to 0.73, while in the three sediment samples, it ranged from 0.66 to 0.67. The Q25/Q75 represents the lower quartile/upper quartile. The Q25/Q75s of eccentricity data of W1, W2, W3, W4, W5, W6, W7, S1, S2, and S3 were 0.64/0.82, 0.61/0.78, 0.60/0.75, 0.60/0.77, 0.60/0.77, 0.61/0.81, 0.62/0.79, 0.61/0.78, 0.61/0.82, 0.54/0.77, 0.54/0.71, and 0.56/0.81, respectively. Moreover, the median solidity of MPs in the nine water samples ranged from 0.85 to 0.93, while in the three sediment samples, it ranged from 0.66 to 0.67. The Q25/Q75s of solidity data of W1, W2, W3, W4, W5, W6, W7, S1, S2, and S3 were 0.79/0.94, 0.86/0.97, 0.75/0.95, 0.88/0.96, 0.86/0.96, 0.81/0.95, 0.78/0.93, 0.77/0.94, 0.69/0.93, 0.81/0.95, 0.84/0.94, and 0.66/0.96, respectively. The shapes of MPs detected in water and sediment samples showed comparable patterns in their distribution. Analyzing the data on eccentricity and solidity, it was evident that long fragments predominantly characterized the MP particles found in this study.

### 3.4. Estimated Daily Intake (EDI) of MPs

Using the determined concentrations of MPs in the simple drinking water system, we estimated the EDIs of these MPs for adults, children, and infants living in a mountainous region in Taizhou, China through the consumption of drinking water, and the results are shown in Table 2. Among the general public, it was found that infants had the highest EDI of MPs, ranging from 2.14 to 31.26 MPs/kg bw/day, followed by children (1.41–20.67 MPs/kg bw/day) and adults (1.05–15.35 MPs/kg bw/day). This indicates that infants may be more vulnerable to exposure to MPs, compared to children and adults, through the consumption of water, likely because of their lower BW and a higher IR of water [63]. Research indicated that the mean levels of PET in infant feces were over ten times higher than those in adults, also illustrating that infants may be more vulnerable to MP occurrence in various kinds of mediums, including drinking water [64].

Taghipour et al. [65] estimated the daily intake of MPs in Zahedan, Iran for children (0.16–15 MPs/kg bw/year) and adults (0.07–5.7 MPs/kg bw/year). Another study indicated that infants consuming treated water in eastern China had an estimated daily uptake of MPs at a rate of 45.5 to 75.0 MPs/kg bw/per day, which is roughly double the intake compared to adults [66]. In this study, the number of MPs ingested by humans through drinking water was significantly higher than in the West Asia region and was close to that of the eastern part of China. This could be attributed to the rapid economic development in China, which has led to the increased use of plastics. Given the well-known health risks induced by MPs, the exposure to MPs through drinking water in remote mountainous areas—where advanced water pretreatment facilities are often lacking—warrants the attention of the government to safeguard the health of the residents in mountainous areas [15,16,17,67]. The EDIs calculated in this study provide baseline data for estimating the overlooked exposure to MPs for people living in mountainous regions with independent drinking water systems.

## 4. Conclusions

To the best of our knowledge, this is the first study looking into the presence of MPs in drinking water in mountainous areas with basic filtering capabilities and independent, uncomplicated drinking water systems. Findings indicated that of the MPs found, the most prevalent polymer types in water samples were PE, PU, and PET, and in sediment samples, the most prevalent polymer types were PVC, PU, and PE. The majority of the MPs found had a size of less than 100 μm. When considering MPs of various polymer kinds in each site as a whole, the length of the pipeline tended to increase the amount of MPs in drinking water. Meanwhile, MPs in drinking water from the reservoir to the tap were not caught by this village’s filtration system. This suggests that in order to prevent MP contamination, higher-standard water treatment systems are needed in rural regions, especially those that are mountainous regions. Fortunately, boiling water can precipitate scales along with MPs, which, to some extent, reduces the quantity of MPs in the water. In general, future studies should concentrate on all individuals living in both rural and urban settings, especially in isolated mountainous places, and offer practical solutions for reducing microplastic contamination and enhancing water purification.

## Figures and Tables

**Table 1:** Lengths of water pipes from various sampling sites to the reservoir’s raw water.

Sampling Sites	Water Pipe Length (m)
W1/W2/W3	0 (Reservoir)
W4	1016
W5/W6	1021
W7	1288
W8	1186
W9	1722

**Table 2:** Estimated daily intakes (MPs/kg bw/day) of MPs through drinking water for adults, children, and infants.

	W1	W2	W3	W4	W5	W6	W7	W8	W9
Adults	2.27	1.69	1.05	3.80	4.52	7.20	6.03	7.72	15.35
Children	3.06	2.27	1.41	5.11	6.09	9.69	8.11	10.40	20.67
Infants	4.63	3.43	2.14	7.73	9.21	14.66	12.27	15.73	31.26

## Data Availability

Data will be made available upon request.

## Supplementary Materials

The following supporting information can be downloaded at: https://www.mdpi.com/article/10.3390/toxics12110807/s1, Table S1: The parameters for the c MP abundance calculation; Table S2: The abbreviations and full names of various types of microplastics; Table S3: The quantities of each MP type identified at each water sampling sites (Number of infrared assessed particles before calculation); Table S4: The quantities of each MP type identified at each sediment sampling sites (Number of infrared assessed particles before calculation); Figure S1: Map of the research location and sampling sites; Figure S2: Flowchart of pretreatment steps for water samples; Figure S3: Flowchart of pretreatment steps for sediment samples; Figure S4: The quality of MPs identified in twelve samples; Figure S5: Schematic diagram of the symbolic meaning of eccentricity and solidity.
