# Supplementary material for: The Detection and Analysis of Microplastics in a Typical Mountainous Drinking Water System in China
Source: Toxics. 2024 Nov 8;12(11):807. doi: 10.3390/toxics12110807 (PMC11598732; doi:10.3390/toxics12110807)
Supplement: Supplementary file 1 [file toxics-12-00807-s001.zip › toxics-3275191-supplementary.pdf]

SUPPORTING INFORMATION

The detection and analysis of microplastics in a typical mountainous drinking water system in China.

Textual supplement material 1: The instrument operation steps for the identification of microplastics.

After the absolute ethanol had completely evaporated, the glass was carefully positioned on the sample holder. The 8700 LDIR system was operated in attenuated total reflection (ATR) mode. Upon the launch of the Agilent Clarity software, the sample holder was mechanically placed into the sample chamber. The software swiftly scanned the designated area at a constant wavenumber of 1800 cm−1 to identify the presence of all particles. Subsequently, it scanned an empty area without particles to serve as the reference background. Continuing the analysis, spectra for all particles within the mid-infrared range (1800-975 cm^{-1}) were obtained. The Agilent Clarity then compared these spectra against a standard spectrum, thereby extracting data on the quantity, polymer type, and dimensions of the particles. The instrument's measurement scope was set between 20 and 500 μm, and only microplastics (MPs) with a match value exceeding 0.65 were counted in the study. Further details on the quantification methodology of the microplastic content are provided in the Supplementary Materials in Textual Supplementary Material 2.

Textual Supplementary Material 2: Microplastic abundance calculations

Specific amounts of water and sediments were pretreated and tested. And the infrared scan was limited to 1h to keep expenses in check. Partial particles were analyzed using the 8700 LDIR.

The microplastic (MP) abundance was measured in MPs/L for water and MPs/kg for sediment (dry weight). The abundance of each MP type was determined using a straightforward equation:

$$\text{Abundance} = \frac{Total\ particle\ count \times Infrared\ assessed\ microplastic\ count}{Infrared\ assessed\ particle\ count \times Sample\ Quantity\ (L\ or\ kg)}$$

Table S1. The parameters for the c MP abundance calculation.

Sampling sites	Sample volume	Total Number of Particles	Infrared assessed particle count
W1	3L	1019	351
W2	3L	935	350
W3	3L	4181	365
W4	2.5L	1740	722
W5	1L	821	410
W6	1L	1567	359
W7	1L	987	320
W8	1L	785	332
W9	1L	1999	326
S1	17.24g	2414	383
S2	15.28g	1489	350
S3	17.56g	1296	330

Table S2. The abbreviations and full names of various types of microplastics.

Full name	Abbreviation
Polyvinylchloride	PVC
Polyethylene terephthalate	PET
Chlorinated polyethylene	CPE
Acrylate copolymer	ACR
Polyurethane	PU
Polyethylene	PE
Polybutylene adipate terephthalate	PBAT
Styrene–butadiene–styrene	SBS
Polymerized styrene butadiene rubber	SBR
Polyvinyl butyral	PVB
Polylactic acid	PLA
Acrylonitrile butadiene styrene	ABS
Butadiene rubber	BR
Ethylene acrylic acid	EAA
Polyoxymethylene	POM
Ethylene vinyl acetate copolymer	EVA
Polypropylene	PP
Polytetrafluoroethylene	PTFE
Polystyrene	PS
Polymethylmethacrylate	PMMA

Table S3. The quantities of each MP type identified at each water sampling site (number of infrared assessed particles before calculation).

Sampling sites	PVC	]	PET	Fluorosilicone rubber	Fluororubber	CPE	ACR	PU	PE	PBAT	SBS	SBR	PVB	PLA
W1	5	-	5	4	5	3	-	1	51	-	-	-	-	-
W2	1	-	4	3	5	-	2	2	42	-	-	-	-	-
W3	-	-	2	-	-	1	1	4	-	-	-	-	-	-
W4	18	1	3	1	4	19	1	55	19	1	1	1	-	-
W5	-	6	12	-	-	2	10	8	9	6	-	1	1	2
W6	2	3	7	-	-	3	9	9	4	2	-	-	-	2
W7	-	-	1	-	2	-	-	2	59	-	-	-	-	-
W8	1	-	8	1	2	1	3	1	87	-	-	-	-	-
W9	-	-	4	2	4	-	2	-	64	-	-	-	-	1

Table S3. The quantities of each MP type identified at each water sampling site (number of infrared assessed particles before calculation, continued).

**Sampling sites**	**ABS**	**Polybutadiene**	**BR**	**Methyl methacrylate–Butadiene–Styrene**	**EAA**	**POM**	**EVA**	**PP**	**PTFE**	**PS**	**Phenolic epoxy resin**	**Phenol formaldehyde resin**	**PMMA**
W1	-	-	-	-	-	-	-	-	-	1	1	1	-
W2	-	-	-	1	-	-	-	-	-	-	-	2	-
W3	-	-	-	-	-	-	-	-	-	-	-	-	1
W4	-	-	1	-	1	1	-	-	-	-	-	2	-
W5	1	-	3	-	1	-	1	-	5	1	-	4	1
W6	-	2	-	-	1	1	-	1	-	8	-	-	-
W7	-	-	-	-	-	-	-	-	-	-	-	-	-
W8	-	-	-	-	-	1	1	-	-	-	-	1	-
W9	-	-	-	-	-	3	-	-	-	-	1	1	-

Table S4. The quantities of each MP type identified at each sediment sampling site (number of infrared-assessed particles before calculation).

Sampling sites	PVC	Polysulfone	PET	Fluorosilicone rubber	Fluororubber	EVA	CPE	ACR	PU
S1	17	-	-	2	-	5	-	-	34
S2	23	-	2	7	1	-	4	2	-
S3	6	1	6	4	1	1	2	-	3

Sampling sites	PE	PTFE	PMMA	PP	PLA	BR	POM	Phenol formaldehyde resin
S1	3	1	-	-	-	-	-	-
S2	26	-	-	-	-	1	1	-
S3	7	1	3	2	1	-	-	1

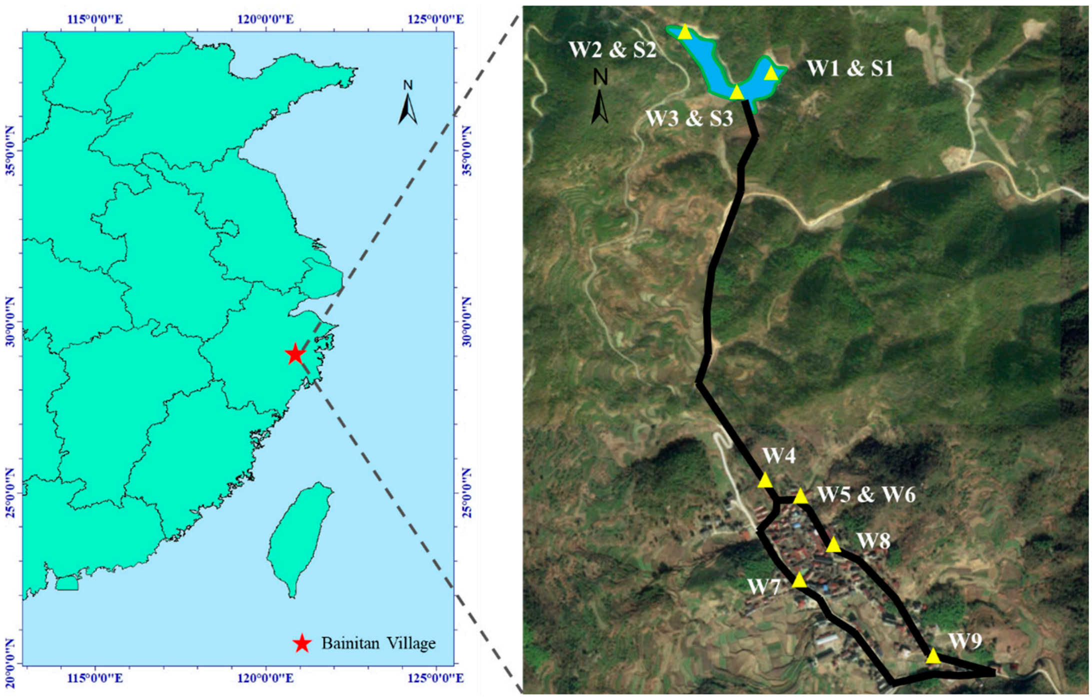

Figure S1. Map of the research location and sampling sites.

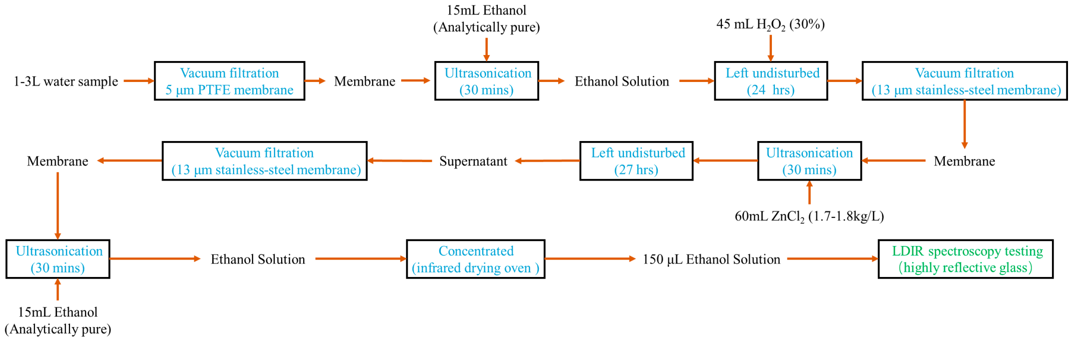

Figure S2. Flowchart of pretreatment steps for water samples.

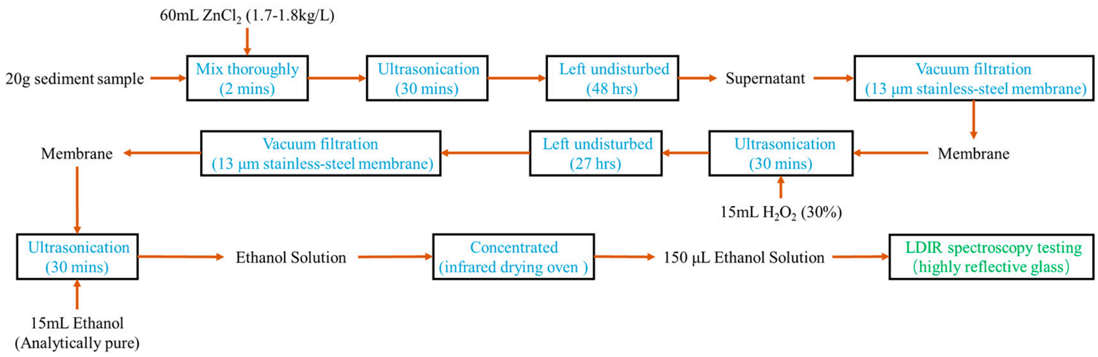

Figure S3. Flowchart of pretreatment steps for sediment samples.

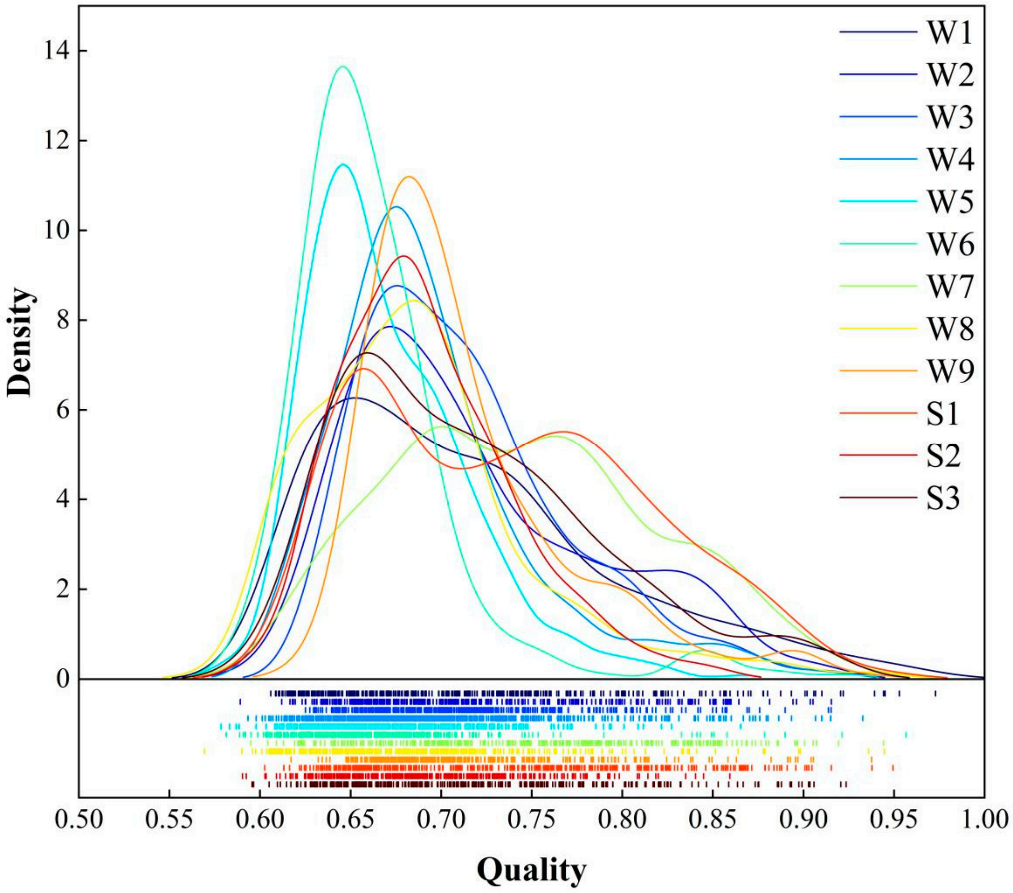

Figure S4. The quality of MPs identified in twelve samples.

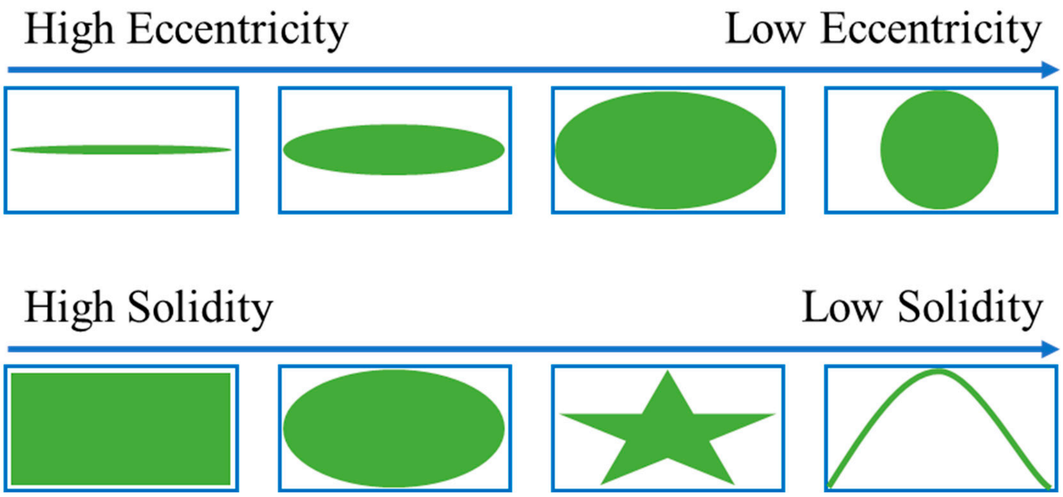

Figure S5. Schematic diagram of the symbolic meaning of eccentricity and solidity.
